# Correction: No turnover in lens lipids for the entire human lifespan

**DOI:** 10.7554/eLife.08186

**Published:** 2015-04-27

**Authors:** Jessica R Hughes, Vladimir A Levchenko, Stephen J Blanksby, Todd W Mitchell, Alan Williams, Roger JW Truscott

Hughes JR, Levchenko VA, Blanksby SJ, Mitchell TW, Williams A, Truscott JW. 2015. No turnover in lens lipids for the entire human lifespan. *eLife* **4**:e06003. doi: 10.7554/eLife.06003

Published 11 March 2015

Due to a conversion error during the production process, the three occurrences of the Greek letter delta (δ) in the following paragraph were mistakenly replaced with the letter ‘ä’:

To ascertain whether the presence of lipids in solution could result in the retention of solvent, a separate batch of samples and blanks was processed with ^13^C-enriched methanol. Solvent mixtures with ^13^C enriched to 10% methanol (corresponding to +900‰ δ ^13^C) were prepared. Lipids were extracted following standard procedures in parallel with unlabeled solvents and their δ ^13^C determined by Isotope Ratio Mass Spectrometry (IRMS). While both δ ^13^C results were in the normal range of −20 to −24‰, a small enrichment was observed at −22.7 ± 0.1 and −21.2 ± 0.7‰ for unlabeled and labeled solvents, respectively. Should these values represent isotopic exchange or retention of solvent by lipids, the carbon weight fraction of this contamination would be ∼0.013% and is therefore negligible.

Additionally, in the following two paragraphs, five occurrences of the Greek letter mu (μ) were mistakenly replaced with ‘ì’:

Chloroform was removed by evaporation in a water bath at ∼50°C followed by drying under vacuum. Copper (II) oxide and silver wire that were previously pre-baked in oxygen were then added to the tubes, which were subsequently flame sealed. Lipids were then combusted in sealed tubes at 900°C overnight. CO_2_ was collected from the breakseals and dried by passing through a cryotrap (−78°C). The amount of CO_2_ was determined and transferred into the small volume graphitization apparatus for graphite target production. ^14^C/^12^C isotopic ratios were measured on the Small Tandem for Applied Research (STAR) accelerator, which has greater than 0.5% precision for samples above 50 μg. Typical sample sizes were in the range of 70–120 μg of carbon. As the small weight of each sample made them susceptible to contamination, blanks that were subjected to the same procedural steps as the lens samples (including extraction steps) were processed with each batch of samples. Blanks produced a residual solvent carbon mass of 10–20 μg following evaporation and were measured for radiocarbon on the Australian National Tandem for Applied Research (ANTARES) accelerator (Fink et al., 2004), which provides greater accuracy for samples less than 50 μg carbon.

Lipid extracts (n = 5) were dried under a stream of nitrogen at 37°C and reconstituted in 100 μl phosphate buffered saline. The amount of protein in each sample was determined using a standard BCA assay as described previously (Smith et al., 1985) and calculated as a fraction of the total amount of lipid in the extract.

The article has been corrected accordingly.

